# Supplementary material for: Mutual Effect of Gypsum and Potassium on Nutrient Productivity in the Alfalfa–Grass Sward—A Case Study
Source: Plants (Basel). 2023 Jun 8;12(12):2250. doi: 10.3390/plants12122250 (PMC10302495; doi:10.3390/plants12122250)
Supplement: Supplementary file 1 [file plants-12-02250-s001.zip › plants-2399596-supplementary.pdf]

Supplement

Table S1. The dependence between the content of nutrients in the sward of the alfalfa–grass mixture and total yield, n = 30.

No.	Trait	Equation	R^2	*p*
1	K1	K1 = 10.3TY – 47.1	0.60	≤ 0.001
2	K2	K2 = 5.45TY + 25.3	0.36	≤ 0.001
3	K3	K3 = 20.7TY – 172.9	0.77	≤ 0.001
4	Mg1	Mg1 = 0.71TY + 1.83	0.83	≤ 0.001
5	Mg2	Mg2 = 0.49TY + 6.36	0.23	≤ 0.01
6	Mg3	Mg3 = 1.56TY + 8.22	0.65	≤ 0.001
7	Ca1	Ca1 = –1.63TY + 50.1	0.13	≤ 0.05
8	Ca2	Ca2 = 2.89TY + 2.85	0.07	ns
9	Ca3	Ca3 = 4.22TY – 30.9	0.30	≤ 0.01
10	Fe1	F1 = 22TY – 116.2	0.48	≤ 0.001
11	Fe2	F2 = 9.26TY – 65.6	0.20	≤ 0.01
12	Fe3	F3= 21.1TY – 101.9	0.57	≤ 0.001
13	Mn1	Mn1 = 4.7TY + 17.6	0.46	≤ 0.001
14	Mn2	Mn2 = 2.35TY + 87.1	0.01	ns
15	Mn3	Mn3 = 8.38TY – 1,27	0.48	ns
16	Zn1	Zn1 = 4.65TY + 35	0.21	≤ 0.01
17	Zn2	Zn2 = –0.73TY + 95.8	0.01	ns
18	Zn3	Zn3 = 17.4TY – 134.5	0.60	≤ 0.001
19	Cu1	Cu1 = 0.35TY + 13.7	0.02	ns
20	Cu2	Cu2 = 0.12TY + 18.8	0.01	ns
21	Cu2	Cu3 = 2.05TY – 8.7	0.62	≤ 0.001

$p < 0.001$, $p < 0.01$, and $p < 0.05$, respectively; ns – non-significant; Legend: K, Mg, Ca, Fe, Mn, Zn, Cu – nutrients; 1, 2,3 – successive cuts; TY – total sward yield.

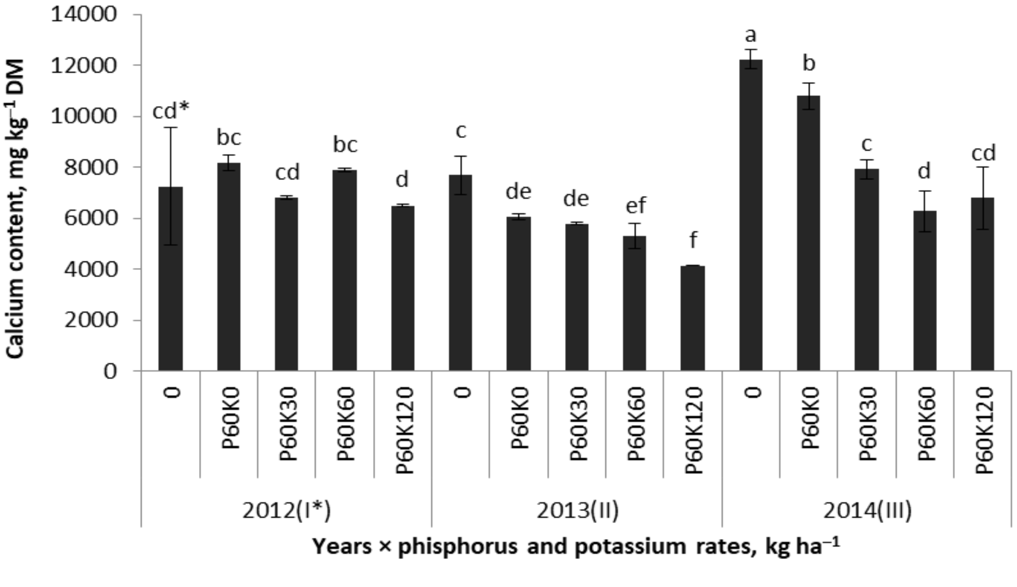

Figure S1. Effect of phosphorus/potassium rates in successive seasons on the content of Ca in the first cut of the alfalfa–grass mixture. [a]Similar letters indicate a lack of significant differences using Tukey's' test. The vertical bar in the column refers to the standard error of the mean; *first main season of the sward use.

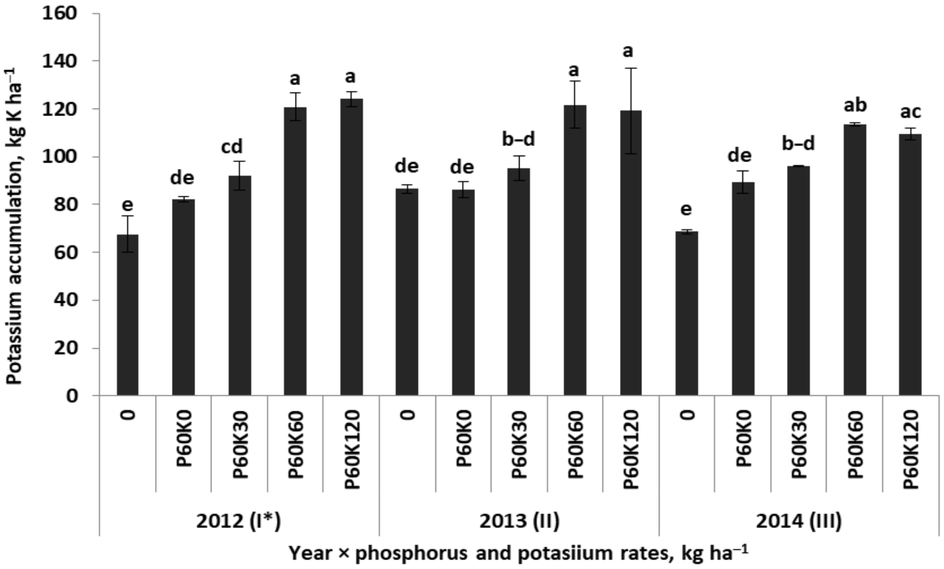

Figure S2. Effect of phosphorus/potassium rates in successive seasons on the accumulation of K in the second cut of the alfalfa—grass mixture. [a]Similar letters indicate a lack of significant differences using Tukey's' test. The vertical bar in the column refers to the standard error of the mean; *first main season of the sward use.

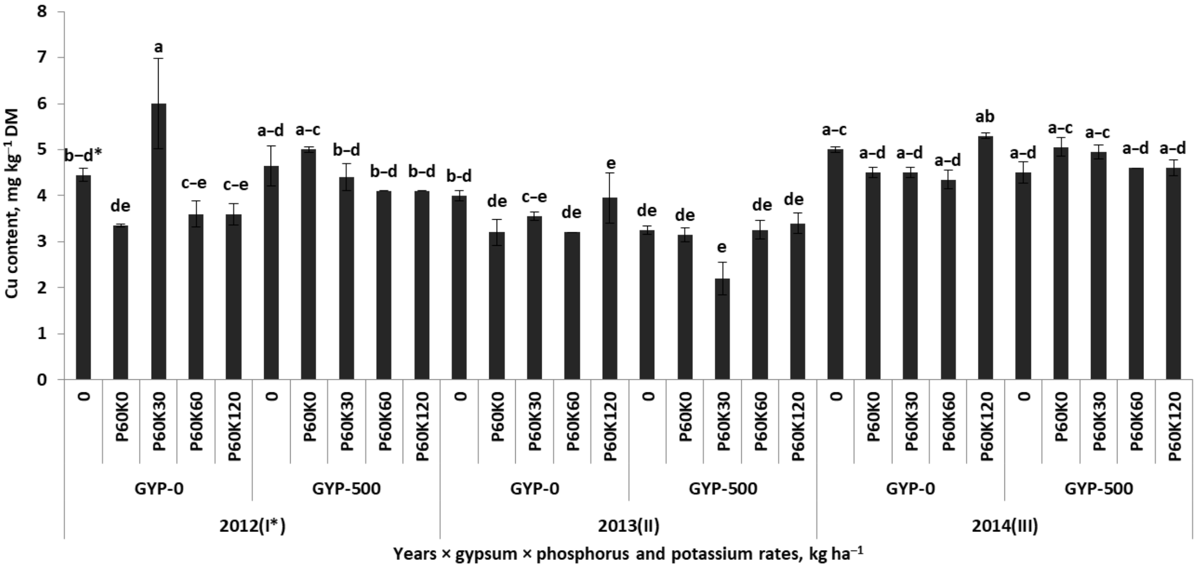

Figure S3. Effect of phosphorus/potassium rates in successive years on the content of copper in the third cut of the sward of the alfalfa–grass mixture. [a]Similar letters indicate a lack of significant differences using Tukey's' test. The vertical bar in the column refers to the standard error of the mean; *first main season of the sward use.

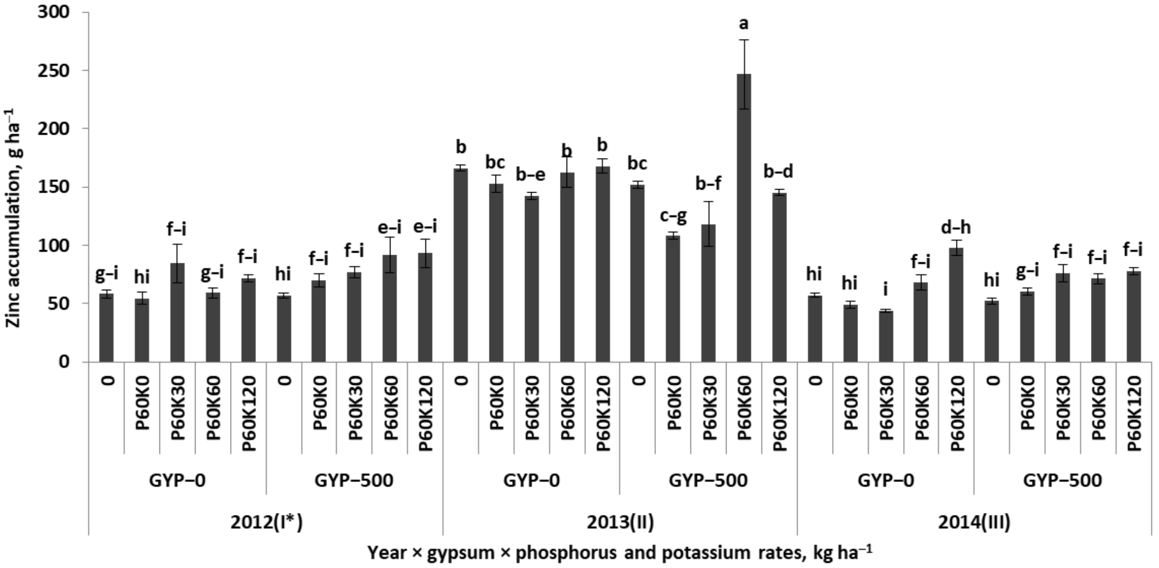

Figure S4. Effect of the interaction of gypsum and phosphorus/potassium rates in successive years on the accumulation of Zn in the third cut of the alfalfa–grass mixture. [a]Similar letters indicate a lack of significant differences using Tukey's' test. The vertical bar in the column refers to the standard error of the mean; *first main season of the sward use.

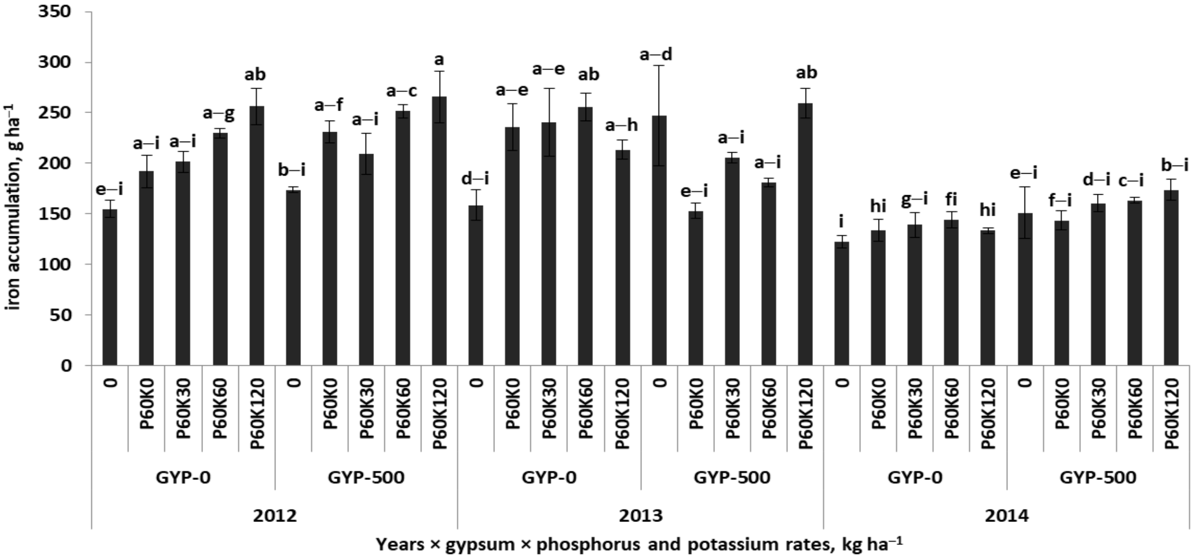

Figure S5. Effect of the interaction of gypsum and phosphorus/potassium rates in successive years on the accumulation of Fe in the second cut of the alfalfa–grass mixture. [a]Similar letters indicate a lack of significant differences using Tukey's' test. The vertical bar in the column refers to the standard error of the mean; *first main season of the sward use.

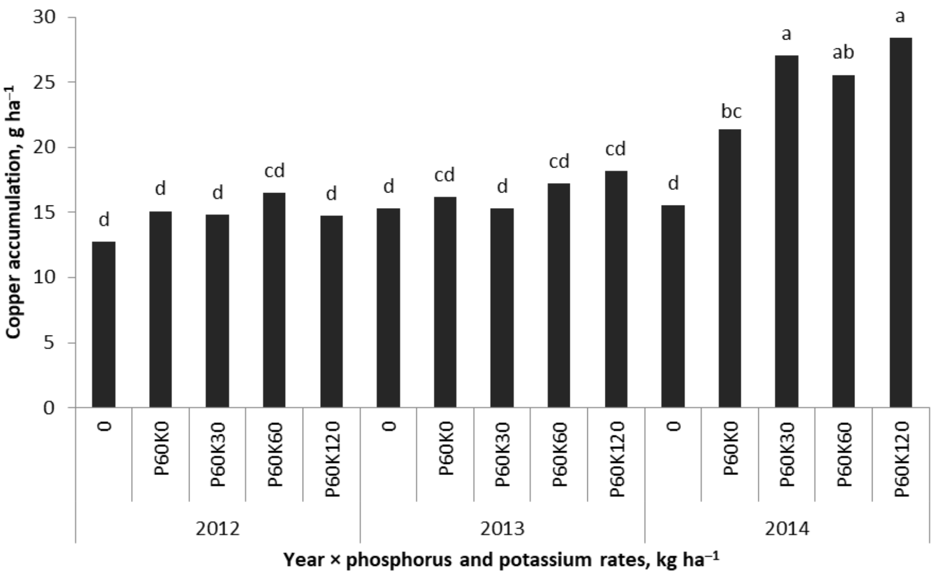

Figure S6. Effect of phosphorus/potassium rates in successive years on the accumulation of copper in the first cut of the alfalfa–grass mixture. [a]Similar letters indicate a lack of significant differences using Tukey's' test. The vertical bar in the column refers to the standard error of the mean; *first main season of the sward use.

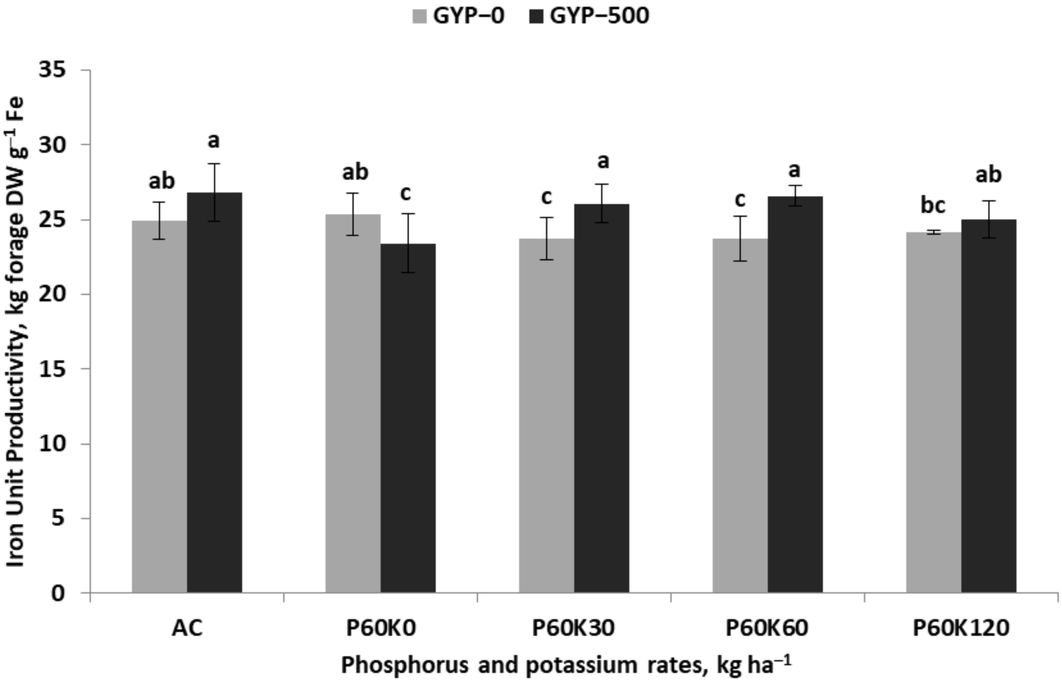

Figure S7. Effect of gypsum and phosphorus/potassium rates on iron unit productivity in the sward of the alfalfa–grass mixture. [a]Similar letters indicate a lack of significant differences using Tukey's' test. The vertical bar in the column refers to the standard error of the mean.

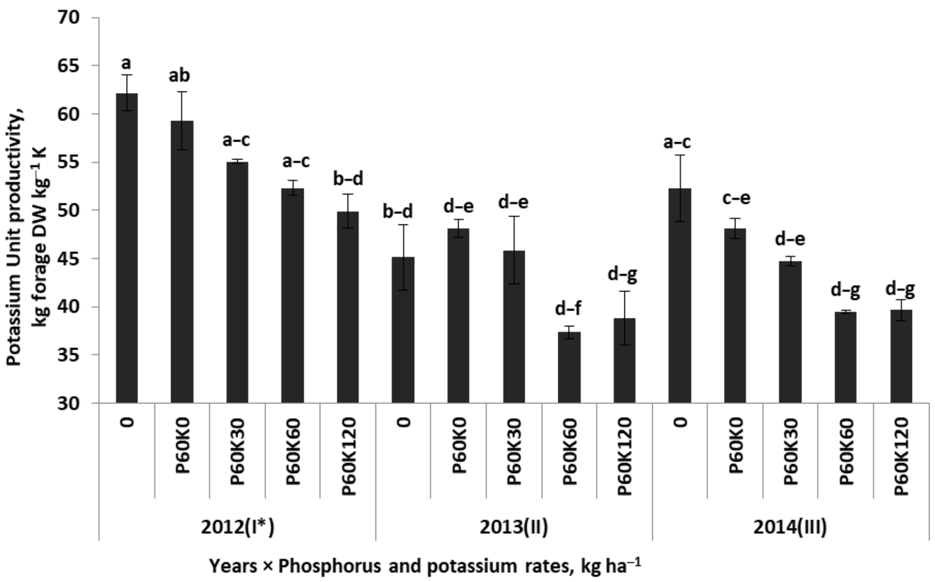

Figure S8. Effect of phosphorus/potassium rates in successive years on potassium unit productivity in the sward of the alfalfa—grass mixture. [a]Similar letters indicate a lack of significant differences using Tukey's' test. The vertical bar in the column refers to the standard error of the mean; *first main season of the sward use.

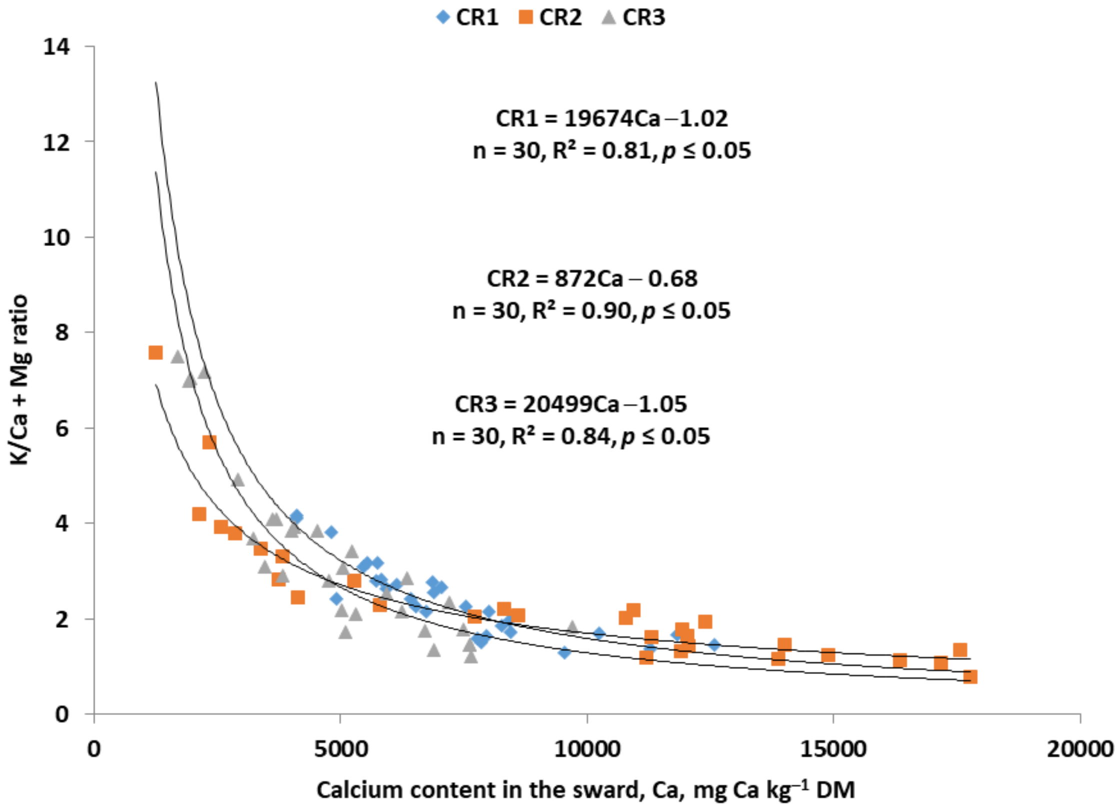

Figure S9. The relationship between the content of calcium and the K/Ca + Mg ratio in the successive cuts of the sward of the alfalfa-grass mixture.
